# Task integration in complex, bimanual sequence learning tasks

**DOI:** 10.1007/s00426-023-01848-2

**Published:** 2023-06-17

**Authors:** Patrick Beißel, Stefan Künzell

**Affiliations:** Institute of Sports Sciences, University of Augsburg, Universitätsstraße 3, 86135 Augsburg, Germany

## Abstract

Sequence learning and multitasking studies have largely focused on simple motor skills, which cannot be directly transferred to the plethora of complex skills found outside of laboratory conditions. Established theories e.g. for bimanual tasks and task integration thus have to be reassessed in the context of complex motor skills. We hypothesize that under more complex conditions, task integration facilitates motor learning, impedes or suppresses effector-specific learning and can still be observed despite partial secondary task interference. We used the Ξ-apparatus to assess the learning success of six groups in a bimanual dual-task, in which we manipulated the degree of possible integration between the right-hand and the left-hand sequences. We could show that task integration positively influences the learning of these complex, bimanual skills. However, the integration impedes but not fully suppresses effector-specific learning, as we could measure reduced hand-specific learning. Task integration improves learning despite the disruptive effect of partial secondary task interference, but its mitigating effect is only effective to some extent. Overall, the results suggest that previous insights on sequential motor learning and task integration can largely also be applied to complex motor skills.

## Introduction

A multitude of tasks which we perform in our everyday lives are accomplished without conscious thought and at most with cursory attention: tying shoelaces, driving cars or typing on keyboards are actions that no longer put a strain on our mental resources, but have become automatic processes. Many of these tasks share two features: firstly, they are often performed by using both hands, for the sake of speed or simplicity. And secondly, they often follow a certain pattern of movement, a motor sequence. In the case of bimanual dual-tasks, two distinct, effector-specific sequences may be implicitly perceived and learned as one single task through the process of task integration (Künzell et al., 2018), further simplifying their use in everyday life. This implicit use of skills results from extensive prior practice of stable motor sequences and not only facilitates daily tasks, but also sets seasoned professionals apart from novices in a variety of settings, such as in the work environment, in music or in sports.

With sequence learning being a central aspect to motor skill acquisition in all facets of life, it has naturally resulted in a huge amount of research on the topic. A groundbreaking study by Nissen and Bullemer (1987), in which participants were asked to perform a dual-task serial reaction time task (SRT task), popularized this research paradigm and has made it a central element of experiments past and present (Ferraro et al., 1993; Shanks et al., 2005; Abrahamse et al., 2010; Brosowsky et al., 2021; for review see Robertson, 2007; Schwarb & Schumacher, 2012, Koch et al., 2018). Since then, the SRT task has been used to investigate many theoretical constructs, such as the locus of sequence learning, the role of multitasking and divided attention (Schwarb & Schumacher, 2012) or the influence of explicit knowledge (Kantak et al., 2012). However, one aspect that most corresponding literature has in common is their focus on fine, simple motor skills, which are of course ideal to study in an experimental environment (Wulf & Shea, 2002; Levac et al., 2019; also e.g. Mayr, 1996; Berner & Hoffmann, 2008, 2009; Schumacher & Schwarb, 2009; Wiestler et al., 2014; Pelzer et al., 2021; Röttger et al., 2021). It must be critically questioned, however, whether conclusions drawn from studies on simple motor skills can be directly transferred to complex motor skills. Key pressing, finger tapping, pointing or similar simple tasks are normally already perfected skills in participants and thus allow for at most minor changes in response to practice (Sternad et al., 2014). They also lack the variability of valid approaches to solve a task that is inherent in more realistic and complex tasks (Levac et al., 2019). Commonly accepted beneficial effects on motor learning gained from manipulation of practice variables might even have an adverse effect in the context of complex motor skills (Wulf & Shea, 2002). The aim of this study is, therefore, to begin with bridging the gap between simple and complex motor skills by building on a foundation of established insight for simple motor skills and using an adapted experimental setup that allows for cautious transfer of insights to complex motor skills.

To this effect, the present experiment seeks to show that the mechanism of task integration is a central factor also in bimanual motor sequence learning for complex motor skills. We furthermore investigate the partial integration of subsequences in the course of motor sequence learning. In the following, we will provide a short overview of the modern perspective on task integration, a note on task complexity in motor sequence learning, as well as a short summary of the study by Schmidtke and Heuer (1997) which has served as a basis for this experiment. We will then shortly outline our adaption of this study.

The task integration hypothesis has its origins in studies by Rescorla and Wagner (1972) who postulated that organisms can detect meaningful relationships between simultaneously occurring events (Rescorla, 1988). This idea was advanced by Reber (1992) who describes implicit learning as a basic ability to incorporate all input from stimulus environments and then focus on true covariations between events, which would then guide further behavior. In the context of multitasking, task integration can be described as explained by Schmidtke and Heuer (1997; Heuer & Schmidtke, 1996) in their influential studies on the subject: as the process of two distinct tasks not being processed separately, but functionally treated as a single task or sequence. Usually, the simultaneous learning of a secondary task is detrimental to performance (Heuer & Schmidtke, 1996; see also for factors influencing impact of secondary task: de Oliveira et al., 2017), leading to dual-task interference due to both tasks competing for limited mental resources, due to a limited central capacity (Schmidtke & Heuer, 1997) or as a consequence of judgment and decision-making (Broeker et al., 2018). But task integration can greatly influence the learning process of dual-tasks. If compatible structures between two tasks can be successfully identified as covariations, then task integration may well be accomplished, benefitting both learning and subsequent performance (de Oliveira et al., 2017; Schmidtke & Heuer, 1997). It can, however, also lead to the adverse effect by attempting to merge two tasks that are too dissimilar to be easily integrated and yet cannot be kept apart (Hazeltine & Schumacher, 2016; Röttger et al., 2021). Röttger et al. (2021) call this inability to distinctly separate individual tasks ‘task confusion’, which entails that learning of a sequence inherent within one task can be severely disrupted when the second task is generated randomly.

There are two predominant theories that can be used to explain this phenomenon, as well as the general workings of task integration in the context of dual-tasks: Logan’s (1988) Instance Theory and the attention hypothesis (Logan & Etherton, 1994), which Pelzer et al. (2021) explain and relate to each other. The Instance Theory is centered around the idea that all input perceived during the processing of a trial, including stimulus and response, is stored in long-term memory as a joint instance or episode. As long as a certain temporal proximity is given, this includes all co-occurrences between information, regardless of actual relevance to the task, as stated by the attention hypothesis. This also overlaps with Reber’s (1992) insights on task integration mentioned earlier. Meaning that even if the trial consists of two distinct movements, the whole performance during the trial, as well as similarities between tasks, are perceived as a single-task set and stored as such in one single instance or episode (Pelzer et al., 2021; Röttger et al., 2021). When the stimuli are repeated, the stored instance is re-activated in order to enable a faster response (Pelzer et al., 2021). If both stimuli of the trial are repeated consistently, then the acquired within-trial associations can consolidate (Röttger et al., 2021). If, however, one of the stimuli differs from the learned pattern, the re-activation of the stored memory episode can result in conflict between what was learned and what is immediately required (Pelzer et al., 2021), thus leading to dual-task costs or task confusion. This process of storing and retrieving instances of sequential motor tasks has been researched in several different areas of motor control and has recently been summarized by Frings et al. (2020) into the Binding and Retrieval in Action Control (BRAC) framework. They postulate that binding and retrieval of event-files are functionally different and distinct processes that are individually influenced by top-down and bottom-up processes, such as attention, instruction, experience, or perception. The event file contains all features found in the learning environment: stimulus, response and resulting effect alike. If any of these features is encountered again, the event file is retrieved. As mentioned earlier, whether the retrieval has beneficial or detrimental effects in any given scenario depends on the congruence of event file and situation. This framework also applies to motor learning involving task integration.

Ultimately, a dual-task sequence can effectively be acquired when both the within-trial and the across-trial co-occurrences can be learned consistently. There is some debate on when said across-trial co-occurrences are learned, however. While Schmidtke and Heuer (1997) assumed that it is acquired in parallel to the within-trial associations, a newer study by Röttger et al. (2021) indicates that when certain criteria are met, the within-trial (or across-task) contingencies are learned first and then connected to successive task pairs through associative chaining. This seems to hold true as long as the two tasks share a close temporal proximity, and no instructions are given which would incentivize a mental separation of the two tasks. Instruction generally may have the potential to shape the perception and thus representation of tasks (Dreisbach et al., 2007; Gaschler et al., 2012; Halvorson et al., 2013; Künzell et al., 2018), although there are findings that indicate the opposite, too (Ewolds et al., 2021). Röttger et al. (2021) conclude that in the event of one stimulus within a dual-task trial being consistently random and thus unpredictable, integration is impossible and in turn hampers across-trial learning as well as implicit sequence learning as a whole. Similar insights were found in several other studies (Keele et al., 2003; Halvorson et al., 2013; Röttger et al., 2019; Zhao et al., 2020). Röttger et al. (2021) go one step further by attributing the root cause of dual-task costs to a lack of across-task predictability instead of parallel response selection processes as indicated by prior research (Schumacher & Schwarb, 2009). While this and many other questions in the field of implicit sequence learning are certainly still in need of further research, we can nevertheless conclude that for task integration to be beneficial in dual-tasks, tasks need to be presented with close temporal proximity and random interference from either task needs to be minimal. In experiments, this should allow for effective across-task and within-task integration, leading to markedly different performance compared to tasks with random interference. This approach is certainly not a novel design and will be used as a basis for this experiment, too. As we argue that this experiment is based on more complex tasks compared to the majority of task integration literature, we will justify our reasoning. The term task complexity seems to be used quite liberally in motor sequence literature and claims to higher complexity tasks in comparison to simple tasks are based on increased motor complexity, longer execution time, a higher rate of errors, more difficult sequences, greater practice requirements or higher demands on attention and memory (Holper et al., 2009; Levac et al., 2019; Verstynen et al., 2005; Du & Clark, 2018). We build upon the most recent definition by Levac et al. (2019) who propose that complex motor tasks, especially real-world tasks, have nested redundancy. They propose that while “redundancy is present when there is a greater number of execution variables than variables that define the result of the task” (Levac et al., 2019, p. 2), the task itself may contain a further level of redundancy. In the context of a task that uses the arm, this entails: intrinsic redundancy, meaning the infinite configurations of the three arm joint angles during task execution; extrinsic redundancy, referring to the strategy or path that is being followed to complete a task; and task redundancy, which describes how precise a task has to be executed to be fulfilled. In the context of traditional SRT tasks centered around pressing a key, redundancy would consequently be rather low, as the fingers would normally be placed directly on the key, which would be pressed down until it triggers a response. Contrary to key-pressing tasks, our task has a greater redundancy and thus complexity, which will be detailed later. With this more complex task, we replicate the experiment of Schmidtke and Heuer (1997) and additionally test the idea that task integration also occurs when correlations are shown not only for complete movement sequences but also for partial sequences.

With their paper on task integration, Schmidtke and Heuer (1997) were among the first to study this mechanism in the context of implicit motor sequence learning. In experiment one, they sought to prove that task integration would essentially negate the detrimental effects of a secondary task if the secondary task’s structure can be incorporated with the primary task, forming an integrated sequence. In contrast, a secondary task with random or unfitting elements would naturally lead to impairment of learning. Participants had to perform a visual SRT task—pressing one of four buttons with index and middle fingers in response to LEDs lighting up—in parallel with an auditory go/no-go task in which they had to respond to high and low tones by pressing a foot pedal or refraining from it. Participants were assigned to the two control groups, a single-task and a random group, or the two treatment groups. While all groups performed the visual SRT task with a sequence of 6 stimuli repeated 15 times, the structure of the secondary task differed. The single-task group did not have a secondary task, yet the other groups had random stimuli or a sequence of five and six tones, respectively. This setup would not allow task integration processes to work for the control groups and group (D-5), but all the more effectively for group (D-6). Group (D-5) was put in place in case of the individual tasks being learned independently, as this group would then have benefitted from its shorter and more frequently repeated secondary task.

All participants completed a single-task practice phase at the start of the experiment, which consisted of two random blocks of each the visual and auditory task. They were assigned to one of the four groups in accordance with their performance and then went through the main practice phase with a total of eight trial blocks. In the following first test phase, they completed two blocks with the familiar sequence, followed by two random blocks and two familiar blocks. Both tasks were randomized in the random blocks. The second test phase was a single-task test of the visual task with three blocks with the pattern familiar—random—familiar. The experiment was concluded with an interview and an anticipation task to test for explicit knowledge in the visual task.

The random catch blocks were meant to assess the degree of acquired sequence knowledge by the increase in reaction times in response to the unfamiliar blocks. Response times for correct responses and error percentages of mistakes or wrong behavior were measured for the visual and auditory task, respectively. Likewise, an implicit learning score was calculated from the response time difference between familiar and random blocks. Also, a certain degree of response conflict between foot and hand was taken into account by additionally focusing on manual responses after auditory go trials only.

Schmidtke and Heuer ultimately found an advantage of group (D-6) over group (D-5) with its different length sequences in the dual-task for both visual and auditory tasks. In regard to sequence learning, both groups also performed better than the random control group in most instances. During the single-task test phase, however, differences between the dual-task groups disappeared, which the authors attributed in part to the fact that half of the learned integrated sequence had been removed. All in all, this led them to the conclusion that while task integration is certainly only one mechanism supporting implicit learning in parallel with others, it can have a strong influence in dual-task situations. In scenarios with a systematic relation between tasks, it can be as beneficial to motor learning as it can be detrimental when there is a random relation.

As Schmidtke and Heuer’s paper on task integration is one of the most influential ones in the field of implicit motor sequence integration, it was an obvious decision to model our own experiment after their example and to adapt it to assess complex motor sequences in a bimanual dual-task. Bimanual dual-tasks have been shown to be a form of multitasking (Swinnen & Wenderoth, 2004) which is suited to the task at hand. Binding strategies have been found in this context that attempt to integrate tasks into a gestalt to overcome coordination constraints. Bimanual movements also increase in complexity and lead to reduced accuracy and stability when non-isodirectional movements are involved (Wenderoth et al., 2002). In order to ensure the use of a complex task, we used an apparatus consisting of two levers that would necessitate the full use of both arms and the shoulders to respond to stimuli, resulting in a high degree of intrinsic redundancy (Levac et al., 2019). While each of the 64 total correct responses required precise final positioning of both levers to be valid, the approach to the end position displayed a high degree of extrinsic redundancy. Each correct response could be approached simultaneously or successively along 3 axes each, thus offering over 720 possible “trajectories” for 1 dual-task response alone. The basis for our data was a SRT task, as it has been shown to be a reliable tool in many similar previous studies (see also Abrahamse et al., 2010; Hazeltine & Schumacher, 2016; Koch et al., 2018; Schumacher & Schwarb, 2009). We measured response times needed for completion of the two simultaneously presented tasks. We chose to present both tasks at the same time as we could then be certain that dual-task costs would inevitably occur (Hazeltine & Schumacher, 2016; Schumacher et al., 2018) which then in turn could potentially be influenced by task integration processes. Simultaneous task presentation also ensured that we measured actual sequence learning and not just the expression of learned sequences, as parallel processing of overlapping dual-tasks disrupts the learning process itself (Schumacher & Schwarb, 2009). Upon completion of one trial, the next appeared instantaneous. We aimed to keep the development of explicit knowledge to a minimum, which is usually required when longer response-to-stimulus-intervals are used (Abrahamse et al., 2010; Destrebecqz & Cleeremans, 2001).

Unlike with Schmidtke and Heuer (1997), our secondary task was also a visual task which required a motor response, following the same modality as the primary task. Labeling the tasks as primary and secondary may in fact be misleading in our case, as participants could choose to perform the tasks in any order. The nature of these task demands allowed us to more effectively trace performance in this dual-task back to the structure of the used sequences.

The respective groups and sequence structures in our experiment were similar to the original design. We decided, however, to also assess whether sequence learning and more specifically task integration would still be beneficial if parts of the secondary task were replaced with random interference. This setup is supposed to mimic a more realistic approximation of motor learning in reality, as movement patterns are not always repeated in the exact same manner or only parts of a sequence may be repeated, such as in dance choreographies.

Our design leads us to assess the following hypotheses in this experiment. The first assumption is that task integration facilitates motor learning with complex bimanual dual-tasks, especially compared to motor sequences with structures unfit for task integration. This has been shown for fine motor skills in previous studies (Berner & Hoffmann, 2008, 2009; Levac et al., 2019; Mayr, 1996; Pelzer et al., 2021; Röttger et al., 2021; Schumacher & Schwarb, 2009; Wiestler et al., 2014; Wulf & Shea, 2002) and can, therefore, be expected to hold true in the present one. Groups with more ideal conditions for task integration should hence perform better than groups with less ideal or impossible conditions. This naturally includes the assumption that underlying sequence structures are being learned and performance improvement cannot solely be attributed to task familiarization.

The second hypothesis is centered around the assumption that task integration in this particular setup is attempting to merge two equally valued tasks into one. Task integration should thus suppress or impede effector-specific learning of the individual tasks in bimanual dual-tasks. This also implies that no one task should be favored over the other. If the tasks were not treated equally, one could expect a notable difference in response time between the participants' hands.

Finally, it can be expected that the process of task integration has adapted to cope with interferences, which motor learning is usually subjected to in realistic circumstances. As such we hypothesize that to a certain degree, task integration allows for partial sequence learning despite random interferences.

## Method

### Participants

In this experiment, 96 participants took part voluntarily or for course credit. The participants’ mean age was 22.3 years (SD 3.04). 52 participants were female and 44 male. Only right-handed people were accepted into the experiment. They were assigned to one of six groups (*n* = 16) based on their performance in the pretest. Group size was determined through a G-Power (Faul et al., 2007) a priori sample size calculation based on effect size (*f = *0.56) of Schmidtke and Heuer’s (1997) test phase 1. Power analysis (1 − *β* = 0.95) indicated a minimum of 15 participants per group, which we increased to 16. Findings of this study should generally be transferable to healthy, non-elderly and non-learning-impaired adults.

### Apparatus

**Fig. 1:**
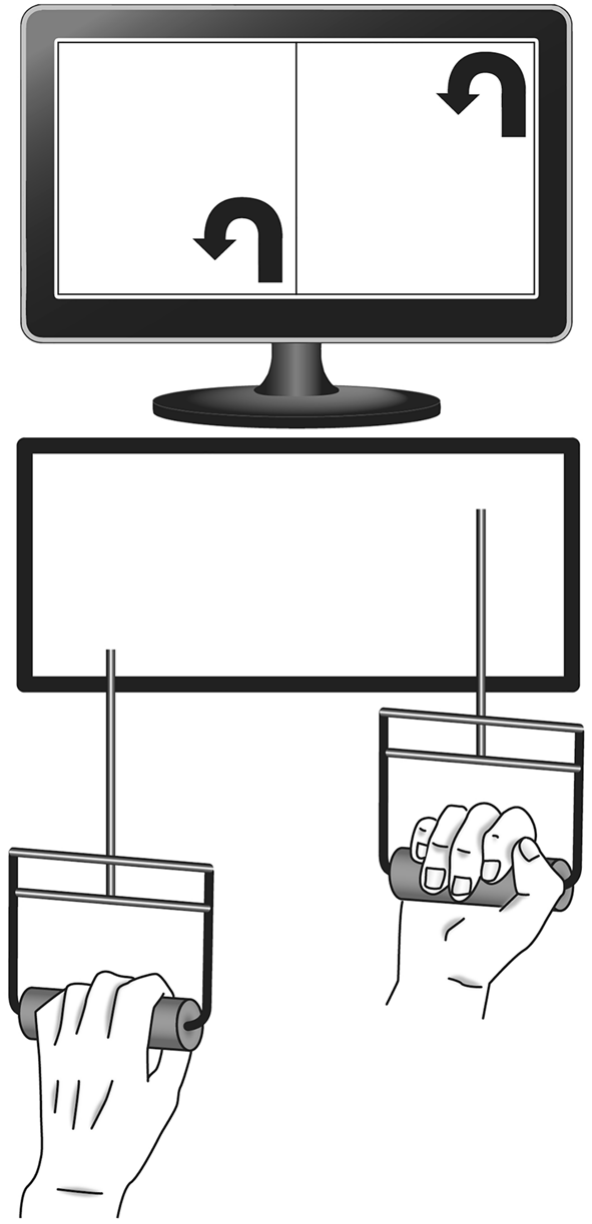
Ξ-apparatus and stimuli presentation. Figure adapted from Hossner and Ehrlenspiel (2010). The apparatus was screwed down to allow for energetic movements. Stimuli could appear in one of four corners of the screen’s respective halves. The arrows’ position indicated where the tip of the handle had to be moved to in the axial and sagittal plane, while the direction indicated how the handle was to be twisted. A video demonstration is available at (https://rb.gy/chbzj8)

Participants were seated in front of a Ξ-apparatus (Ξ read as “csi” for complex sequencing inventory; see Fig. 1) and a computer screen. The Ξ-apparatus was modeled after the one used in a study by Hossner and Ehrlenspiel (2010) and consisted of two parallel, vertical levers which were gripped with either hand and could independently be either pushed forward or pulled back along the horizontal axis. The handles could also be both tilted and twisted inwards or outwards to mechanical stops. Each lever dimension had to be set to its maximal extent for any completion attempt to be counted as valid. The bimanual manipulation of these levers within the possible six degrees of freedom allowed for a total of 2^6^ = 64 unique lever positions or elements. Stimuli corresponding to these elements (see Fig. 1) appeared on the screen in front of the participants and were immediately replaced with new stimuli as soon as the prior one was correctly set. Note that in this experimental design, no error could occur, because the response time only ends when the participants have set the levers correctly in all dimensions. The stimuli always appeared in pairs, each of the adjacent symbols corresponding to the participants’ left and right hands. The response time (RT), consisting of reaction time and movement time, was consistently measured and recorded in a.csv file after completion of each block. Extreme differences in response times between groups due to anything other than sequence structure were minimized through initial tests, as well as identical setup and similar tasks.

### Transparency and Openness

We report how we determined our sample size, all data exclusions (none excluded), all manipulations and all measures in the study. All data and code used for data collection are available at (https://rb.gy/chbzj8). Data were analyzed using Jamovi, version 1.6.23 (The Jamovi Project, 2021). This study’s design and its analysis were not pre-registered.

### Design and Procedure

**Fig. 2:**
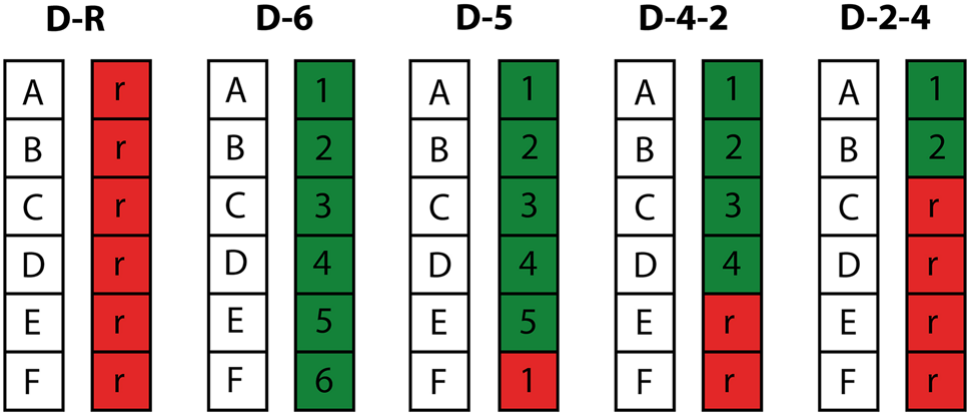
Task structure for dual-task groups (*n* = 16). Identical right-hand task indicated by letters ‘**A**–**F**’, repeating lever positions of the left-hand task by numbers and random lever positions by the letter ‘r’. Each dual-task block consisted of ten sequences (60 trials) during the practice phase and five sequences (30 trials) during the test phase. Group D-5 started the next sequence with the successor of the last action performed, here with action no. 2. Groups (not shown) did not have a left-hand task

Each participant in one of the six respective groups performed two serial RT tasks simultaneously, which had similar sequence structures: on their right hand, all participants practiced one task, a sequence of six elements which repeated over the course of a block. The groups are set apart by the structure of the elements corresponding to the participants’ left hands in the other task. Two groups functioned as control conditions, a single-task group (s) with no left-hand sequence and the dual-task group (D-R), with no sequence, but random elements on the left-hand side. Both conditions made any task integration process impossible by default, while still allowing for improvement of RT through learning of the underlying right-hand sequence. The experimental conditions were designed as follows (see Fig. 2): group (D-5) had a sequence of five elements on the left hand, parallel to the one with six elements on the right. This ensured that task integration processes would not apply yet learning of the hand-specific sequences was still possible. Group (D-6) was given ideal task integration conditions with a six-element sequence on either hand, with fixed element pairs in every repetition. The remaining two groups, group (D-4-2) and group (D-2-4), meant to assess partial sequence learning in spite of random interferences, were designed similarly to group (D-6), but after four and two fixed element pairs, respectively, the remaining two and four left-hand elements were replaced with random elements before repetition of the sequence.

The experiment took place over the course of 2 consecutive days, during the same time of day and under the same conditions. Before the experiment, participants were informed that they would participate in an experiment assessing reaction times while performing a bimanual task and were instructed to always act as quickly as possible. This was followed by a short introduction to the Ξ-apparatus and the stimuli. They then had to complete a pretest, which consisted of all 64 possible combinations in a random order, and were asked to complete this practice block in under 300 s. If they succeeded within no more than five attempts, the participants were admitted to the experiment and assigned to their respective groups in accordance with their number of attempts in the pretest in order to create homogenous groups.

Once assigned to a group, the practice phase began. It consisted of 6 blocks of 60 stimuli, each block containing a fixed sequence of 6 element pairs repeated 10 times. Participants received a break of 20 s between blocks, with exception of the final break between block five and six, which was 5 min long. Schmidtke and Heuer (1997) noted a drastic improvement after a 5-min break in their experiment, which we thus implemented, too. This concluded the first day of the experiment.

On the second day, participants were asked to react to random element pairs in order to reacquaint them with the Ξ-apparatus until they signaled readiness. They were told that the procedure would be similar to the day before and then completed the test phase. This phase consisted of 6 blocks with 30 stimuli, starting and ending with 2 blocks each following the practiced pattern and 2 blocks in the middle which were utterly random. Breaks between blocks were all 20 s long.

After the test phase, participants were asked to complete three single-task blocks, using only their dominant right hand. These 3 blocks each consisted of 30 elements and were separated by 20 s breaks. While the second block was once more random, the first and third block followed the individual pattern to which the participants had been exposed in all prior non-random blocks.

The experiment concluded with a short interview, a recognition test and an anticipation test in order to determine the degree of explicit awareness. During the interview, participants were asked whether they had noticed anything notable during the experiment. Further questions to assess extent of awareness were asked if key words such as “pattern”, “order”, “sequence”, “repetition” or similar descriptions were reported. Participants were then also asked to describe the sequence they perceived as accurately as possible.

In the following recognition test, participants were first informed that there had been an underlying sequence in the previous tasks—without being told any specifics—and were then shown a mixture of familiar and unfamiliar element pairs in a random order. They were then asked to label them as familiar or unfamiliar. Participants were allowed to set the levers of the Ξ-apparatus to the shown position if they were so inclined. The respective choices were documented.

In the final anticipation test, participants were reminded that each stimulus of their sequence was naturally followed by a fixed successor. Participants were shown a random part of their sequence and asked to adjust the levers accordingly. They then had to move them into the position which had always followed after this particular stimulus. Participants could act without time constraints and removed their hands from the levers when they had adjusted them in accordance with their prediction. The chosen lever positions were then recorded. This was repeated for all parts of the sequence in a random order. This concluded the experiment.

For both these tests on the control groups, only the performance of the right hand was further analyzed for group (D-R), while group (s) was naturally tested with an adapted single-task setup.

## Results

**Fig. 3:**
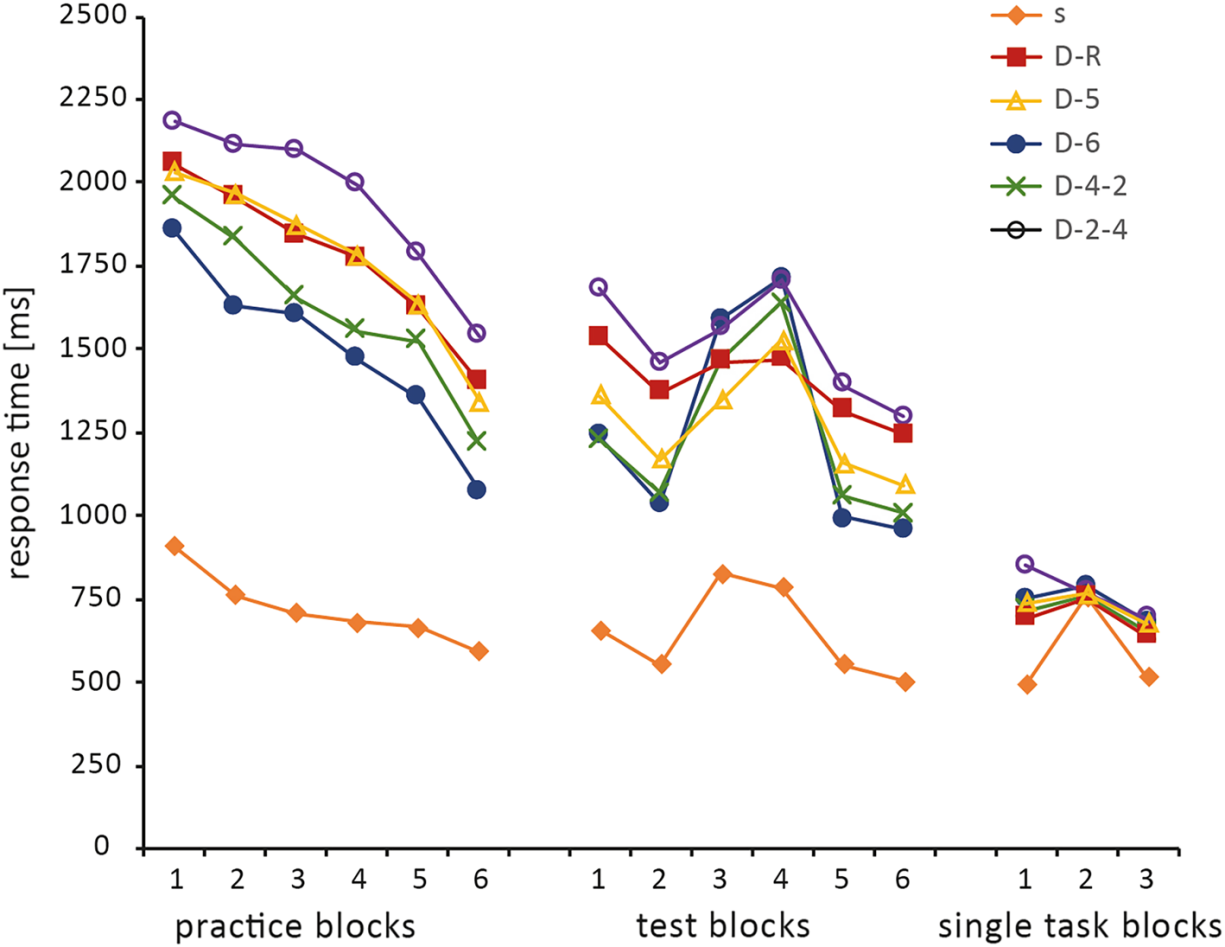
Overview: mean response times per group and block. Means of median RTs of the bimanual SRT task and unimanual single SRT task. The same block structure was used for most blocks, only test blocks 2 and 3 and single-task block 2 were fully random. Breaks between blocks were 20 s long, except for the break prior to the last practice block, which was 5 min. For description of groups (*n* = 16), see text and Fig. 2

In general, participant’s individual response times (RTs) in respective blocks were summarized in medians to reduce the influence of outliers. Statistical group analyses were then performed using the means of participants’ RTs in the individual blocks. Also, the single-task group was calculated separately from all dual-task groups in most instances, as it performed with distinctly lower response times, as had to be expected. Figure 3 provides an overview over the most important results. Prior to statistical analysis, we evaluated the recognition test, anticipation test and interview. The main purpose of these tests was to gauge the extent of influence of explicit knowledge. A participant was considered as having obtained explicit knowledge if the underlying sequence was reported during the interview in any manner, more than half of the recognition test’s items were labeled correctly and three out of six lever positions of the learned sequence were set correctly during the anticipation test. Only a total of seven participants met these criteria, four of them in group (s), one in (D-6) and two in (D-4-2). For the remaining 89 test participants, clearly expressible explicit knowledge can be ruled out and performance generally can be attributed to implicit knowledge.

The first test we calculated was a baseline comparison to check whether participants had successfully been assigned to equally capable groups and to rule out performance differences not resulting from practice (Fig. 3). To avoid any effects of sequence repetitions and ensure similar task conditions, we analyzed the dual-task groups’ RTs of the first sequence within the first practice block—similar to the approach of Künzell et al. (2016) in the analysis of an implicit pursuit tracking task. A one-way between-groups Kruskal–Wallis analysis of variance (ANOVA) with the dependent variable ‘response time’ and the grouping variable ‘group’, (χ^2^ (4) = 8.86, *p = *0.065) showed that the differences in the means between the groups were not large enough to be significantly evident, with RTs (SD) of 2460 (1056) ms, 2419 (1084) ms, 2459 (1452) ms, 2197 (757) ms and 2595 (1127) ms, for groups (D-R), (D-5), (D-6), (D-4-2) and (D-2-4), respectively.

Next, we assessed overall learning of the five dual-task groups during the practice phase by comparing performance of the first and final practice blocks in a mixed ANOVA with the within-subject factor ‘block’ (1^st^ block, 6^th^ block) and the between-subject factor ‘group’ (D-R, D-5, D-6, D-4-2, D-2-4). This showed a significant main effect of ‘block’ with *F* (1, 74) = 248.8, *p < *0.001, *η*^2^_part_ = 0.77 and a significant main effect of ‘group’ with *F* (5, 74) = 3.46, *p = *0.007, *η*^2^_part_ = 0.19, but no significant block x group interaction effects with *F* (5; 74) = 0.8, *p = *0.55, *η*^2^_part_ = 0.05, indicating successful learning but no overall differences owed to the specific group conditions. For a more in-depth look at learning success of the individual groups, we followed up with repeated measures within-group ANOVAs with the factor ‘block’. All groups displayed a significant effect of practice on response times with *F* (5, 75) = 103, *p < *0.001, *η*^2^_part_ = 0.87 for group (s), *F* (5, 75) = 46.2, *p < *0.001, *η*^2^_part_ = 0.76 for group (D-R), *F* (5, 75) = 36.6, *p < *0.001, *η*^2^_part_ = 0.7 for group (D-5), *F* (5, 75) = 50.5, *p < *0.001, *η*^2^_part_ = 0.77 for group (D-6), *F* (5, 75) = 36.5, *p < *0.001, *η*^2^_part_ = 0.7 for group (D-4-2) and *F* (5, 75) = 51.9, *p < *0.001, *η*^2^_part_ = 0.78 for group (D-2-4). Post hoc tests further revealed that all groups consistently showed significant improvement between the last two blocks (*p < *0.001), after the 5-min break. Earliest successful learning could be shown for groups (s) and (D-6), with highly significant differences (*p < *0.001) in their RT reduction (SD) between the first and second block of practice with 143.5 (15.0) ms and 227.5 (53.1) ms, respectively. The other groups displayed lower RT reduction in the beginning with 124.9 (60.6) ms for (D-4-2), 98.1 (49.1) ms for (D-R), 68.1 (48.2) ms for (D-2-4) and 61.7 (58.9) ms for (D-5). The latter groups reached a significant difference to the first block after the third (groups (D-4-2) and (D-R)) or forth (groups (D-2-4) and (D-5)) block.

The analysis of the practice phase concluded with a comparison of performance in the final practice block to assess whether any group’s performance had surpassed the others’. To this end, we used a one-way between-groups ANOVA with the dependent variable ‘response time’ and the grouping variable ‘group’, *F* (4, 75) = 7.40, *p < *0.001, *η*^2^_part_ = 0.28 and post hoc comparisons using Tukey’s HSD test. The group with the shortest RT (SD) was (D-6) with 1075 (281) ms, while the groups with the longest RTs were (D-R) with 1400 (254) ms and (D-2-4) with 1549 (282) ms. Group (D-6) was significantly faster than these groups (*p = *0.011 and *p < *0.001, respectively). No significant difference could be shown between the other groups, with the exception of (D-4-2), which was faster (*p = *0.003) than (D-2-4) but did not differ from the other groups. The difference between (D-6) and (D-5) interestingly approached significance (*p = *0.052).

We began assessing the results of the test phase by checking the experiment’s basic hypothesis of groups displaying slower RTs upon being confronted with random blocks instead of familiar ones. We compared the means of RTs in the random blocks to the corresponding RTs of the familiar preceding and succeeding blocks using within-group-dependent samples t tests, either Student’s or Wilcoxon signed rank test. The difference between the first familiar blocks and the random intervention was clearly significant (*p < *0.001) for most groups with the exception of (D-2-4) with *p = *0.044 and (D-R) which showed no significant difference with *p = *0.72. In comparison to the succeeding blocks, the performance of all groups was significantly slower (*p < *0.001) in the random intervention. The confirmation of this basic hypothesis allowed us to continue with a between-group comparison of the extent of implicit knowledge acquired. To this extent, we calculated an implicit learning score (ILS) in a similar manner as Schmidtke and Heuer (1997) did in their experiment, by subtracting the RT means of the two familiar blocks flanking the random blocks from the two random blocks’ RT means. The resulting scores were compared in a one-way between-groups ANOVA with the dependent variable ‘implicit learning score’ and the grouping variable ‘group’, *F* (5, 41) = 17.5, *p < *0.001, *η*^2^_part_ = 0.68 and post hoc comparisons using the Games-Howell post hoc test. The single-task group was included in this comparison. As can be seen in Table 1, the group displaying the highest value and thus having learned most effectively was (D-6), followed by (D-4-2). The lowest score was shown by the random control group (D-R).

**Table 1:** Implicit learning score: test phase dual-task

Group	*N*	Mean	SD	SE
s	16	251	77.9	19.5
D-R	16	123	104.2	26.1
D-5	16	274	155.0	38.8
D-6	16	638	208.3	52.1
D-4-2	16	487	276.4	69.1
D-2-4	16	207	132.9	33.2

As can be seen in Table 2, the post hoc test further showed that most differences between groups were significant, with a few notable exceptions. Clear differences could be shown for the two control groups, (s) and (D-R), in comparison to most treatment groups. Only (D-5) showed no difference to (s). Also, (D-2-4) was not statistically different to the control groups, as well as to (D-5). Among the treatment groups, a definite trend in learning success can be outlined: group (D-6) as the most successful group was markedly better than all other groups, except for the second most effective group, (D-4-2). The latter group displayed a higher score than the control groups and (D-2-4), but not than (D-5). (D-5) showed to have learned more than the random control group. Finally, group (D-2-4) registered a surprisingly low score which did not set it apart from the control conditions.

**Table 2:** Games–Howell post hoc test: implicit learning score–test phase dual-task

		s	D-R	D-5	D-6	D-4-2	D-2-4
s	Mean difference	–	128**	− 23.4	− 387***	− 236*	44.0
	*p* value	–	0.006	0.994	< 0.001	0.041	0.859
D-R	Mean difference		–	− 151.1*	− 515***	− 364**	− 83.7
	*p* value		–	0.035	< 0.001	0.001	0.377
D-5	Mean difference			–	− 364***	− 213	67.4
	*p* value			–	< 0.001	0.116	0.772
D-6	Mean difference				–	151	431.3***
	*p* value				–	0.514	< 0.001
D-4-2	Mean difference					–	280.0*
	*p* value					–	0.016
D-2-4	Mean difference						–
	*p* value						–

*p < 0.05, **p < 0.01, ***p < 0.001

For the final single-task test phase, we followed the same strategy as before. We started with a baseline comparison to test for preliminary differences, then checked for differences between the random and fixed blocks within group. Finally, we compared the implicit learning scores that resulted from this phase. Group (s) was naturally included in this analysis, as it served as the control group for this test phase. For the baseline test, we calculated a between-groups Kruskal–Wallis ANOVA with the dependent variable ‘response time’ and the grouping variable ‘group’, which found significant interaction (*χ*^2^ (5) = 42.2, *p < *0.001). We, therefore, followed up with pairwise comparisons, which revealed that while group (s) was markedly different (*p < *0.001) to every other group, all other groups were on a comparable level. This result could already be expected from the groups’ mean RTs (SD) with 494 (66) ms, 693 (129) ms, 738 (113) ms, 751 (197) ms, 717 (97) ms and 851 (326) ms, for groups (s), (D-R), (D-5), (D-6), (D-4-2) and (D-2-4), respectively. However, the former dual-task groups were not fully equal, as a Bayesian ANOVA with the dependent variable ‘response time’ and the grouping variable ‘group’ indicated (BF_01_ = 1.89).

The differences between the random and fixed parts of the test phase were calculated by comparing the random test block with the mean of the familiar blocks through within-group dependent samples Student’s paired t-tests. The tests revealed highly significant differences for most groups (*p < *0.001), with the exception of (D-R) which was nevertheless significant (*p = *0.005) and (D-2-4) which was not (*p = *0.095). Calculating an implicit learning score is therefore still a viable approach. Group (D-2-4) was still included in further analysis as a point of comparison but should be regarded cautiously.

The single-task ILS was calculated similarly to the dual-task one, by subtracting the mean RT in the practiced blocks from the RT of the random block. A between-groups Kruskal–Wallis ANOVA with the dependent variable ‘response time’ and the grouping variable ‘group’ was used to compare ILS scores, again followed by pairwise comparisons. A significant interaction (*χ*^2^(5) = 40.4, *p < *0.001) was found. The mean RTs (SD) were 252.5 (65) ms for (s), 89.7 (109.5) ms for (D-R), 60.6 (41) ms for (D-5), 71.6 (59) ms for (D-6), 73.5 (57) ms for (D-4-2) and, as indicated by the previous t test, -4.0 (170) ms for (D-2-4). The post hoc comparisons revealed significant interaction between group (s) and every other group with *p = *0.002 with (D-R) and *p < *0.001 with every other group. Other than that, no significant differences between any of the other groups can be reported. These results are mirrored by Bayesian ANOVAs with the dependent variable ‘response time’ and the grouping variable ‘group’ for all groups (BF_10_ = 8.42e + 6) and with the single task group excluded (BF_01_ = 1.34).

We furthermore investigated additional aspects of the experiment for our second hypothesis and as a proof of concept. The performance of each individual dual-task group with their respective left and right hands in test and practice phase was compared within group to assess whether changes of strategy may have taken place at certain points during the experiments and to estimate the extent of potential effector-specific learning. This should likewise allow for insight into the effective application of task integration processes. In this regard, we also compared the response times of the partial-sequence groups (D-4-2) and (D-2-4) in repeating parts to those in random parts of their trials to assess whether task integration had made sequence learning possible despite the random interferences. Finally, the aforementioned interview, recognition test, and anticipation test should allow for an estimate on the degree of influence of explicit knowledge.

For an initial overview of differences in performance by hand, the means of hand-specific RTs in all blocks were compared using separate within-group repeated measure ANOVAs with the dependent variable ‘response time’ and the grouping variable ‘hand’ for both practice and test phase of all dual-task groups. No significant interaction could be found for practice or test phase, not for the control group (D-R) (F (1; 30) = 1.36, *p = *0.25, *η*^2^_part_ = 0.04; F (1; 30) = 0.64, *p = *0.43, *η*^2^_part_ = 0.02), nor for the treatment groups (D-5) (*F* (1; 30) = 0.5, *p = *0.48, *η*^2^_part_ = 0.02; *F* (1; 30) = 0.01, *p = *0.92, *η*^2^_part_ = 0.0003), (D-6) (*F* (1; 30) = 1.80, *p = *0.67, *η*^2^_part_ = 0.06; *F* (1; 30) = 0.19, *p = *0.66, *η*^2^_part_ = 0.006), (D-4-2) (F (1; 30) = 0.46, *p = *0.5, *η*^2^_part_ = 0.02; *F* (1; 30) = 0.01, *p = *0.89, *η*^2^_part_ = 0.0003) or (D-2-4) (*F* (1; 30) = 0.05, *p = *0.81, *η*^2^_part_ = 0.002; F (1; 30) = 0.12, *p = *0.74, *η*^2^_part_ = 0.004). We furthermore calculated post hoc tests to assess each individual block for differences between the hands to see whether one effector could have been dominant at some point, yet no significant differences could be found for any of them.

More diverse results could be found for the comparison of the fixed parts of groups (D-4-2) and (D-2-4) with their randomized counterparts. To be able to check for successful learning of a partial sequence despite random interference, we compared the mean RTs of the fixed parts of a block with the random ones, assuming that the acquired sequence would be performed faster than the random portion. Within-group repeated measure ANOVAs with the dependent variable ‘response time’ and the grouping variable ‘structure’ were used on practice and test blocks of both groups, excluding the fully random blocks of the latter phase. This revealed significant differences for (D-4-2) during the practice phase (*F* (1; 30) = 11.0, *p = *0.002, *η*^2^_part_ = 0.27) and the test phase (*F* (1; 30) = 14.4, *p < *0.001, *η*^2^_part_ = 0.32) alike. While this paints a rather clear picture in regard to our third hypothesis, the results for (D-2-4) were less consistent. While displaying a significant difference during the practice phase (F (1; 30) = 4.7, *p = *0.038, *η*^2^_part_ = 0.14), no difference could be found for the second phase (F (1; 30) = 2.16, *p = *0.152, *η*^2^_part_ = 0.07).

## Discussion

The approach taken to assess the effects of task integration in this experiment is based on previously established experimental designs (Schmidtke & Heuer, 1997) and equipment (Hossner & Ehrlenspiel, 2010). Yet as this approach has not been used before in the context of complex, bimanual motor sequence learning, several measurements were taken as a proof of concept and to ensure the validity of our results. We deem this necessary as the findings of studies on simple motor skills cannot simply be generalized for complex motor skills (Wulf & Shea, 2002) and as such, methods should be critically evaluated, too. Not only did we carefully monitor participant recruitment and group assignment, but we also compared the groups’ RTs in the first sequence of practice as a baseline to ensure that all groups would start out on a similar level. Our results confirm that no dual-task group displayed significantly faster RTs at the beginning of the practice phase. Furthermore, we wanted to ensure that learning was taking place and RT reductions were due to learning of the task-inherent sequences and not due to increased familiarity with the set up. The overall analysis of the practice phase showed a significant reduction of RTs, yet no significant differences owed to group design between groups. The within-group comparisons of the practice blocks showed successful RT reductions for all groups, the results emulating those of Schmidtke and Heuer (1997), as the greatest RT drop was registered for the last block after the 5-min break. The distinct increase in performance due to this slightly longer break indicates that it may be used for subliminal consolidation of the practiced movement patterns. Studies in the area of motor memory consolidation have also reported short-term performance increases after 5-min breaks for motor sequence learning (e.g. Brawn et al., 2010; Hotermans et al., 2006), attributing them to either an activated state of the motor memory or simply dissipation of fatigue after practice (Heuer & Klein, 2003; Hotermans et al., 2006). The participants also showed significantly slower RTs during the random blocks of the test phase, except for the two groups with the least learning success, groups (D-R) and (D-2-4). This fact strongly suggests that RT reductions can be mostly attributed to learning of the motor sequence.

Furthermore, we wanted to ensure that the tasks had been executed and—if possible—learned as an integrated compound sequence, excluding alternative strategies such as primarily focusing on one effector or serial completion of the bimanual task. These could lead to results similar to studies with uncorrelated sequences and thus perceivable differences (Berner & Hoffman, 2008). We, therefore, compared the effector-specific RTs in every block with each other. As no differences could be found, we can assume that the effector-specific tasks were treated as a compound sequence by all dual-task groups and can be interpreted as such.

Finally, the interview as well as recognition and anticipation test were meant to estimate the degree of implicit and explicit knowledge in participants. Our aim was to keep explicit knowledge to a minimum to keep conditions comparable to Schmitdke and Heuer’s study (1997), but we did not aim to assess the influence of either, especially as with regard to the process purity problem (Schumacher & Schwarb, 2009), boundaries are difficult to discern. Implicit sequence knowledge might also be independent from explicit awareness anyway (Song et al., 2007). As most participants can safely be considered to have no explicit knowledge of the underlying sequences and the three dual-task participants that do are also not confined to one group, we concluded that the influence of explicit knowledge can be disregarded for this experiment and no exclusion of data was necessary.

As our experiment has thus shown to have been built on a solid foundation, we now turn to our hypotheses.

For our first hypothesis, we tried to show that task integration facilitates motor learning with complex bimanual dual-tasks in a situation that permits effective task integration. This assumption has been shown to hold true for simple motor tasks (see also Berner & Hoffmann, 2008, 2009; Schumacher & Schwarb, 2009; Levac et al., 2019; Pelzer et al., 2021; Röttger et al., 2021) but has not been conclusively shown for complex motor skills. Successful learning of the dual-task in general, regardless of underlying learning mechanism, can be shown if within-group performance at the end of the practice phase is markedly better than in the beginning, as well as through increased response time during the random intervention of the test phase. If task integration enables improved learning, however, performance of groups with a task structure more suited to task integration should overall result in shorter response times as well as higher implicit learning scores than in those groups with less suited structures. As such, group (D-6) should be superior to the other groups, as its structure allows for within-task and across-task learning, followed by (D-4-2) and (D-2-4) with the same structure but random interference. The performance of (D-5) should be worse than the aforementioned groups, as across-task integration has been made impossible. If this group performed on a similar level, however, learning success could be entirely attributed to within-task learning instead of task integration. Also, the random group (D-R) should be on an equal or inferior level to (D-5).

Our results suggest that task integration processes can facilitate sequence acquisition, as they strongly resemble the results of Schmidtke and Heuer’s experiment one. During the practice phase, learning was most effective in groups (s) and (D-6) and especially for the latter group, a significant RT improvement could be shown earlier than in the original experiment. In a similar fashion, every group displayed a significant improvement after the 5-min break, which seems to allow for subliminal consolidation of the sequences. The between-group comparison at the end of the practice phase already indicated the results of the test phase with (D-6) being clearly faster than the slowest groups, (D-R) and (D-2-4). Although distinct differences could not be shown for the other groups, the respective RTs were in line with the group order proposed in the base hypothesis, the obvious exception being (D-2-4). This pattern then clearly manifested itself again through the implicit learning scores of the test phase. The highest scoring dual-task groups were those with ideal or close to ideal conditions for task integration, (D-6) and (D-4-2), while the random control group displayed the lowest score. Interestingly, (D-5) scored markedly higher than the random group, which indicates that other learning mechanisms work in parallel to the automatic tendency of attempting to integrate two tasks into a single-task set (Pelzer et al., 2021; Schmidtke & Heuer, 1997) and hints at partially effective learning of within-task contingencies. Implicit learning in general was still hampered due to impossible across-task predictability (Roettger et al., 2021). The group which defied expectations most, however, was (D-2-4) which performed on a level similar to (D-R), despite having the opportunity for limited task integration. This can certainly be traced back to the group’s task design, which will be further discussed for the third hypothesis, yet already shows that tasks than can be partially integrated are not necessarily easier to learn. Nevertheless, we can conclude that tasks which are ideal for task integration facilitate performance in comparison to conditions which do not. This finding is consistent with those of other studies for fine motor tasks (see e.g. Schumacher & Schwarb, 2009; Schwarb & Schumacher, 2012; de Oliveira et al., 2017) and can thus be expected to hold true for complex motor tasks as well. As the less suited groups perform worse, one could also argue that across-task integration seems to be the driving mechanism behind successful task integration and that a lack of proper across-task integration might be the primary source of dual-task costs, as has been proposed in recent literature (Roettger et al., 2021; Zhao et al., 2020). This raises the question about the extent of influence across-task integration and in extension task integration have over motor learning in general.

If we assume task integration to be a natural and dominant mechanism in this context (Pelzer et al., 2021; Roettger et al., 2021; Zhao et al., 2020), it should have distinct ramifications on effector-specific learning, leading us to our second hypothesis: task integration should suppress or impede effector-specific learning of the individual tasks in bimanual dual-tasks. To assess this, we had our participants perform a single-task test phase after their dual-task test phase. If all groups had performed on a similar level, the random intervention would not have been significantly different, and the implicit learning score had thus been low, it would have confirmed our hypothesis outright. This would also have indicated that across-task integration was the sole and dominant determinant of task integration. Across-task integration might, however, not be of central importance or alternative mechanisms such as a focus on within-task learning might be favored as a tool more fitting the circumstances. It is worth keeping in mind that resources can be allocated unevenly to different tasks (Broeker et al., 2021; Schmidt & Dolis, 2009; Strayer & Drews, 2007; Wickens et al., 2015) and as such, a change of strategy to focus on more manageable tasks (Broeker et al., 2021) is to be expected. If that were the case, then groups with less ideal task integration conditions should have had an advantage in the single-task tests, as they might prioritize one task and thus produce two task sets instead of one integrated set, as shown in previous studies (Halvorson et al., 2013; Schumacher & Schwarb, 2009). Our results strongly suggest a middle ground between these antithetical assumptions: while the control group (s) was clearly superior in this phase, no significant differences could be found between any other group in the baseline test and the ILS comparisons. Similar results have been reported by Schmidtke and Heuer (1997) who attribute the equal performance of their groups to removal of response conflict and of elements from a learned integrated sequence. These same arguments apply in this context. Comparisons of within-group effector-specific performance have also shown no differences between left and right side for all groups in all blocks, making alternative strategies such as primarily focusing on one side unlikely, even for the random control group. Nevertheless, learning seems to have taken place as significant interaction could be shown between the random and practiced blocks of every group except for (D-2-4). This shows that alternative learning mechanisms, possibly within-task learning, are not fully suppressed and are effective enough to lead to better than average performance. This is consistent with findings from past studies, among others (Bapi et al., 2000; Verwey & Clegg, 2005; Verwey & Wright, 2004) by Berner and Hoffmann (2008, 2009) who have shown that effector-specific learning takes place after extensive practice of fine motor, bimanual SRT tasks. Yet our inherent tendency to utilize task integration processes (see e.g. Pelzer et al., 2021) still seems dominant enough to hamper alternative processes in groups with less ideal conditions, while a single task might not be easily removable from the integrated task set (Freedberg et al., 2014; Hazeltine & Schumacher, 2016; Pelzer et al., 2021; Roettger et al., 2021) for groups with ideal conditions. Also, regarding the co-occurrence of across-trial and within-trial learning during task integration assumed by Schmidtke and Heuer (1997) or the precedence of across-task learning proposed by Roettger et al. (2021), our results suggest that while the latter theory seems to mostly hold true as it explains our groups’ equal performance, some degree of within-task learning still seems to take place. We can, therefore, conclude that task integration processes do not suppress effector-specific learning in the context of complex motor tasks, but certainly impede the acquisition of the effector-specific task, as has likewise been shown for small motor tasks.

We have shown so far that task integration, depending on the task structures, can support or impede sequence learning and that it impedes, yet does not suppress effector-specific learning. But it is still unclear whether task integration will remain to be effective as long as there are parts that can be integrated between two tasks or whether there is a limit to it being beneficial. In everyday situations, motor sequences are rarely encountered without disturbances or task-irrelevant information, so the human mind should be able to adapt to these circumstances, at least to some extent. We can correctly expect distractions to impede the learning process, especially if the distraction and the task are very different in nature (Hemond et al., 2010; Keele et al., 2003)**. However, minor distractions might be mitigated, as the mind can focus on structured parts of tasks, which in turn frees up resources (Broeker et al., 2021). Distractions might even be beneficial if similar processes are engaged (Hemond et al., 2010), yet the reliance on shared resources might also have the opposite effect, too (Hemond et al., 2010; Schumacher & Schwarb, 2009). Considering these findings, we hypothesized that task integration allows for partial sequence learning despite random interferences. We expected group (D-4-2) to perform better than all groups except (D-6), with (D-2-4) only being inferior to these two groups. As we have established with the comparison of left and right effector, task integration can be assumed to be the predominant mechanism at work and differences in performance should thus be mainly attributed to its degree of effectiveness. The comparison of the fixed and random parts of the tasks for group (D-4-2) showed the predicted outcome, as did its performance in comparison to the other groups: the structured and integrated parts of the dual-task were consistently performed faster than the random ones during both the practice and test phase, while overall, it was the second fastest group. We can thus conclude that the parts of the sequence which were suitable for integration were recognized and successfully integrated despite the random interferences. But (D-2-4) defied most expectations. A significant difference between the fixed and random parts could be shown for the practice phase, but not for the test phase. Furthermore, the group generally performed worse than all other groups, even worse than or on par with (D-R). This might be explained through successful retrieval of memory files (Frings et al., 2020; Pelzer et al., 2021) of the structured parts between the tasks, indicated by the significant difference during the practice phase, and the futile attempt to integrate these with the remaining random trials. This attempt would naturally demand more—ultimately wasted—resources, leading to worse performance. The heightened resource demand may stem from the increased task complexity or the activation of other processes trying to facilitate the task (Hemond et al., 2010). The difference’s disappearance in the test phase hints at unsuccessful consolidation attempts over night or simply not enough structure to create a lasting memory episode or instance in the first place.

The disparate results for groups (D-4-2) and (D-2-7) confirm the well-known fact that task integration does not universally improve sequential learning but can have the opposite effect as well (Halvorson et al., 2013; Keele et al., 2003; Röttger et al., 2019, 2021; Zhao et al., 2020). It also confirms our hypothesis as it shows that task integration can still be effective and beneficial despite random interference within the tasks, however, not universally. While trials fit for integration still seem to be recognized despite heavier interference, the resulting attempts to integrate those can lead to costs which outweigh the benefits. With our groups facing either little or much interference disturbing task integration, a finer assessment trying to evaluate a more precise threshold for still manageable interference would certainly be of interest for future research.

## Conclusion

The current research on sequential learning and task integration covers a wide variety of research questions and many theories are still being discussed or improved upon, yet most studies derive their findings from studies on simple motor skills. These cannot reliably be transferred to complex skills, which are relevant to countless activities in everyday life, in music or in sports. It was, therefore, the aim of this study to transfer and critically question previous insights into task integration to complex motor skills. Our assessment of task integration in complex skills has shown that it is indeed a dominant mechanism which can improve the learning of sequences as well as impede it, depending on the task structure. It also appears to impede the acquisition of effector-specific knowledge, while not suppressing other, independent learning processes. And finally, task integration can adapt to effectively compensate random interferences during sequence acquisition, yet only to a certain degree. While this study provides some insight into the role of task integration in the learning of complex motor sequences, more studies on topics surrounding sequential motor learning in this field are urgently needed, as they would make the practical implications derived from this area of study more applicable and accessible to everyone outside the laboratory.

## Data Availability

Study data can be accessed online (Beißel & Künzell, 2019) at (https://rb.gy/chbzj8).
